# Neoadjuvant Stereotactic Body Radiotherapy After Upfront Chemotherapy Improves Pathologic Outcomes Compared With Chemotherapy Alone for Patients With Borderline Resectable or Locally Advanced Pancreatic Adenocarcinoma Without Increasing Perioperative Toxicity

**DOI:** 10.1245/s10434-021-11202-8

**Published:** 2022-02-07

**Authors:** Colin S. Hill, Lauren M. Rosati, Chen Hu, Wei Fu, Shuchi Sehgal, Amy Hacker-Prietz, Christopher L. Wolfgang, Matthew J. Weiss, Richard A. Burkhart, Ralph H. Hruban, Ana De Jesus-Acosta, Dung T. Le, Lei Zheng, Daniel A. Laheru, Jin He, Amol K. Narang, Joseph M. Herman

**Affiliations:** 1 Department of Radiation Oncology and Molecular Radiation Sciences, Johns Hopkins University School of Medicine, Sidney Kimmel Cancer Center, Baltimore, MD USA; 2 University of South Carolina School of Medicine, Columbia, SC USA; 3 Philadelphia College of Osteopathic Medicine, Philadelphia, PA USA; 4 Department of Surgery, New York University Grossman School of Medicine, New York, NY USA; 5 Department of Surgery, Zucker School of Medicine at Hofstra/Northwell, Lake Success, NY USA; 6 Department of Surgery, Johns Hopkins University School of Medicine, Baltimore, MD USA; 7 Department of Pathology, the Sol Goldman Pancreatic Cancer Research Center, Johns Hopkins University School of Medicine, Baltimore, MD USA; 8 Department of Oncology, Johns Hopkins University School of Medicine, Baltimore, MD USA; 9 Radiation Medicine, Zucker School of Medicine at Hofstra/Northwell, Lake Success, NY USA

## Abstract

**Background:**

Patients with borderline resectable pancreatic cancer (BRPC) or locally advanced pancreatic cancer (LAPC) are at high risk of margin-positive resection. Neoadjuvant stereotactic body radiation therapy (SBRT) may help sterilize margins, but its additive benefit beyond neoadjuvant chemotherapy (nCT) is unclear. The authors report long-term outcomes for BRPC/LAPC patients explored after treatment with either nCT alone or nCT followed by five-fraction SBRT (nCT-SBRT).

**Methods:**

Patients with BRPC or LAPC from 2011 to 2016 who underwent resection after nCT alone or nCT-SBRT were retrospectively reviewed. Baseline characteristics were compared, and the propensity score with inverse probability weighting (IPW) was used to compare pathologic/survival outcomes.

**Results:**

Of 198 patients, 76 received nCT, and 122 received nCT-SBRT. The nCT-SBRT cohort had a higher proportion of LAPC (53% vs 22%; *p* < 0.001). The duration of nCT was longer for nCT-SBRT (4.6 vs 2.9 months; *p* = 0.03), but adjuvant chemotherapy was less frequently administered (53% vs 67.1%; *p* < 0.001). Adjuvant radiation was administered to 30% of the nCT patients. The nCT-SBRT regimen more frequently achieved negative margins (92% vs 70%; *p* < 0.001), negative nodes (59% vs 42%; *p* < 0.001), and pathologic complete response (7% vs 0%; *p* = 0.02). In the multivariate analysis, nCT-SBRT remained associated with R0 resection (*p* < 0.001). The nCT-SBRT cohort experienced no significant difference in median overall survival (OS) (22.1 vs 24.5 months), local progression-free survival (LPFS) (13.5 vs. 15.4 months), or distant metastasis-free survival (DMFS) (11.7 vs 16.3 months) after surgery. After SBRT, 1-year OS was 77.0% and 2-year OS was 50.4%. Perioperative Claven-Dindo grade 3 or greater morbidity did not differ significantly between the nCT and nCT-SBRT cohorts (*p* = 0.81).

**Conclusions:**

Despite having more advanced disease, the nCT-SBRT cohort was still more likely to undergo an R0 resection and experienced similar survival outcomes compared with the nCT alone cohort.

**Supplementary Information:**

The online version contains supplementary material available at 10.1245/s10434-021-11202-8.

Pancreatic adenocarcinoma remains an aggressive malignancy with dismal long-term survival outcomes and is expected to become the second leading cause of cancer death by the year 2030.^1,2^ Outcomes are poor, as roughly half of patients present with evidence of metastatic disease at diagnosis. The majority of patients without metastatic disease present with extra-pancreatic extension and involvement of key peri-pancreatic vasculature, rendering complete surgical resection with negative margins challenging.

Nevertheless, response rates for nonoperative therapies have improved, enabling a higher proportion of patients with localized disease to undergo complete resection compared with historical data.^3–8^ Indeed, multi-agent systemic regimens, such as FOLFIRINOX (FFX) and gemcitabine combined with nab-paclitaxel (GnP), which were first demonstrated to improve survival in the metastatic setting, have since been administered in the localized setting with encouraging outcomes.^9–11^

Additionally, advances in radiation technologies have dramatically improved the precision of treatment delivery for pancreatic tumors. One example is the use of the hypo-fractionated stereotactic body radiation therapy (SBRT), in which precisely delivered high doses of radiation per fraction allows treatment during a much shorter interval, usually five fractions or less.^12,13^

In the setting of candidacy for surgical exploration, neoadjuvant radiation can be administered with the goal of increasing the likelihood of margin-negative resection and decreasing the risk of postoperative local recurrence. Either chemoradiation (CRT) or SBRT can be used in this setting, but the latter has value in minimizing the interval between the end of chemotherapy and surgical exploration and in reducing the risk of acute toxicity, treatment-related lymphopenia, and quality-of-life decrement.^14–16^

Nevertheless, the additive benefit of radiation beyond neoadjuvant chemotherapy alone for patients with borderline resectable pancreatic cancer (BRPC) or locally advanced pancreatic cancer (LAPC) undergoing surgical exploration has not been well-characterized to date because existing data are limited by the lack of inclusion of modern multi-agent systemic regimens and radiation technology.^17–19^ This report describes the long-term pathologic and survival outcomes in addition to the 90-day perioperative toxicity for a cohort of patients treated at single high-volume institution who underwent subsequent surgical resection after receiving neoadjuvant chemotherapy (nCT) alone or neoadjuvant chemotherapy followed by SBRT (nCT-SBRT).

## Methods

### Patient Population and Treatment Course: Neoadjuvant Chemotherapy and SBRT

With institutional review board approval, all patients with BRPC and LAPC diagnosed between 2011 and 2016 at our institution who were subsequently explored for surgical resection after nCT or sequential nCT-SBRT were retrospectively reviewed. Patient eligibility and follow-up evaluation are discussed in Appendix 1. Systemic therapy was prescribed at the discretion of the treating medical oncologist. Robust patients with good performance status generally received induction multi-agent chemotherapy such as modified FOLFIRINOX (mFFX) or non-modified FFX, GnP, or gemcitabine, docetaxel, and capecitabine (GTX).

During induction therapy, the patients were serially examined at 2- to 3-month intervals with a pancreatic protocol computed tomography scan to assess chemo-responsiveness and confirm continued treatment eligibility. Although standard practice was to offer SBRT after chemotherapy, if patients had a robust response to chemotherapy with limited vessel involvement, they sometimes underwent surgery without SBRT. For those who received nCT-SBRT, our institutional practice regarding SBRT has previously been described in detail (Appendix 2).^12^

### Clinical Characteristics and Treatment Outcomes

Chemotherapy received after SBRT and before surgery in the nCT-SBRT cohort was included in the calculation of neoadjuvant CT duration. After surgery, pathologic outcomes were centrally reviewed for tumor grade, margin status, and nodal clearance by two dedicated pathologists who re-reviewed the specimens. Pathologic complete response (pCR), was defined as no residual viable tumor, whereas near pCR (npCR) was defined as single cells or rare small groups of cancer cells.^20^ Partial response was defined as residual cancer with evident regression but more than single cells or rare small groups of cancer cells.^20^ Absent response was assigned when extensive cancer was present with no evident tumor regression.^20^

Perioperative toxicity was collected for 90 days after surgery, and complications were graded according to the Claven-Dindo classification.^21^ Perioperative toxicity was compared between cohorts with the chi-square test or Fisher’s exact test. Statistical analysis of survival measures, including overall survival (OS), progression free-survival (PFS), local PFS (LPFS), and distant metastasis-free survival (DMFS) and of how covariates and outcomes were compared by cohort are summarized in Appendix 3.

## Results

### Clinical Demographics and Treatment Characteristics

Retrospective review identified 198 consecutive patients with BRPC or LAPC who met our inclusion criteria. The baseline clinical demographics are summarized in Table 1. Of the 198 patients, 76 (38.4%) received nCT and 122 (61.6%) received nCT-SBRT. No significant difference in age, gender, performance status, tumor location, or CA 19-9 level was observed at diagnosis or post-nCT (all *p* > 0.05). However, the nCT-SBRT cohort had a significantly higher percentage of LAPC patients (53.3% vs 22.4%; *p* < 0.05).

**Table 1:** Clinical demographics

	Chemotherapy *n* (%)	Chemotherapy and SBRT *n* (%)	*p* Value < 0.05
*n*	76	122	
Median age: years (range)	63.9 (40.3–83.2)	63.5 (39.7–83.6)	
Gender			
Female	33 (43.4)	58 (47.5)	
Male	43 (56.6)	64 (52.5)	
ECOG PS			
0	22 (29.0)	48 (39.3)	
1–2	27 (35.5)	73 (59.8)	
Not reported	27 (35.5)	1 (0.8)	
Tumor location			
Head/neck/uncinate	59 (77.6)	82 (67.2)	
Body/tail	17 (22.4)	40 (32.8)	
NCCN staging			< 0.001
BRPC	59 (77.6)	57 (46.7)	
LAPC	17 (22.4)	65 (53.3)	
Median CA 19-9 level (range)			
Baseline	122.5 (9.2– 1552.6)	190.8 (< 1.0–14,004.2)	
After nCT	54.4 (< 1.0–913)	32.5 (< 1.0 –2851.4)	
CT agent			
FFX	43 (56.6)	66 (54.1)	
GnP	11 (14.5)	19 (15.6)	
Other	22 (28.9)	37 (30.3)	
Median induction CT duration: months (range)			
Total duration: months (range).	2.9 (0.7–16.8)	4.6 (0–16.5)	0.029
≥ 4 Months duration	23 (31.1)	74 (62.2)	< 0.001
Adjuvant therapy			
CT	51 (67.1)	64 (52.5)	0.001
Median CT duration: months (range)	4.0 (0–18.5)	2.1 (0–6.0)	0.003
Radiation	23 (30.3)	N/A	N/A

SBRT, stereotactic body radiation therapy; ECOG PS, Easteron Cooperative Oncology Group performance status; NCCN, National Comprehensive Cancer Network; BRPC, borderline resectable pancreatic adenocarcinoma; LAPC, locally advanced pancreatic adenocarcinoma; CA 19-9, cancer antigen 19-9; FFX, FOLFIRINOX; GnP, gemcitabine and nab-paclitaxel; CT, chemotherapy

Regarding chemotherapy type, mFFX was the most commonly administered chemotherapy regimen in both cohorts (56.6% nCT vs 54.5% nCT-SBRT), whereas GNP was the second regimen of choice (14.5% nCT vs. 15.6% nCT-SBRT). The type of chemotherapy administered by cohort did not differ significantly, but the total induction chemotherapy duration differed significantly between the nCT and nCT-SBRT cohorts (2.9 vs 4.6 months; *p* = 0.029). In the nCT-SBRT cohort, 74 patients (62.2%) received more than 4 months of induction chemotherapy compared with 23 patients (31.1%) in the nCT cohort (*p* < 0.001).

Regarding adjuvant therapy, systemic chemotherapy was more frequently administered after surgery in the nCT cohort (67.1% vs 52.5%; *p* < 0.001), and for longer durations (4.0 vs 2.1 months; *p* = 0.003). Notably, 23 patients (30.3%) in the nCT cohort also received adjuvant radiation after surgery for high-risk features, namely, margin positivity in 14 patients (61%) and/or node-positive resections in 20 patients (87%).

### Pathologic Outcomes After Neoadjuvant Therapy

Pathologic outcomes were significantly better in the nCT-SBRT cohort than in the nCT-alone cohort (Table 2). Margin sterilization was achieved for 112 patients (91.8%) in the nCT-SBRT cohort compared with only 53 patients (69.7%) in the nCT cohort (p < 0.001). Only 32 patients (42.1%) were pathologically node-negative in the nCT cohort compared with 72 patients (59.0%) in the nCT-SBRT cohort (*p* = 0.028). Notably, none of the patients in the nCT-alone cohort achieved a pCR, but nine patients (7.4%) in the nCT-SBRT cohort achieved a pCR (*p* = 0.001). The multivariate analysis with propensity score analysis based on IPTW using the propensity score demonstrated that receipt of SBRT still was significantly associated with negative margins, but not for nodal clearance after accounting for potential confounding (Table 3). Vasculature reconstruction was less frequently required in the nCT cohort (20 patients [26.3%]: 18 BRPC patients [30.5%] and 2 LAPC patients [11.8%]). In the nCT-SBRT cohort, 49 patients (40.2%) required reconstruction, including 23 BRPC patients (40.3%) and 26 LAPC patients (40.0%). Among the nCT patients, the median fibrosis of the resected tumor was 35% (range, 0–99%) compared with 70% (range, 0–100%) among the nCT-SBRT cohort.

**Table 2:** Pathologic outcomes

	Chemotherapy (*n* = 76) *n* (%)	Chemotherapy and SBRT (*n* = 122) *n* (%)	*p* Value < 0.05
Margin status			0.0001
Negative	53 (69.7)	112 (91.8)	
Positive	23 (30.3)	10 (8.2)	
Nodal status			0.0279
Node-negative	32 (42.1)	72 (59.0)	
Node-positive	44 (57.9)	50 (41.0)	
Pathologic response			0.001
Complete response	0 (0.0)	9 (7.4)	
Nearly complete response	12 (15.8)	38 (31.1)	
Other	64 (84.2)	75 (61.5)	

SBRT, stereotactic body radiation therapy

**Table 3:** Propensity score-adjusted multivariable analysis

	OR	95% CI	*p* Value < 0.05
Surgical margin			
ATT	0.82	0.69–0.97	**0.0203**
ATE	0.82	0.72–0.94	**0.004**
Pathologically nodal-negative			
ATT	0.93	0.76–1.13	0.4487
ATE	0.92	0.78–1.09	0.3403
Overall survival			
ATT	0.96	0.62–1.50	0.8741
ATE	0.95	0.66–1.38	0.7977
Progression-free survival			
ATT	1.04	0.72–1.51	0.8269
ATE	1.1	0.79–1.54	0.5682
Local progression-free survival			
ATT	0.8	0.55–1.16	0.2332
ATE	0.84	0.59–1.19	0.322
Distant metastasis-free survival			
ATT	1.23	0.84–1.79	0.2843
ATE	1.26	0.90–1.77	0.185

OR, odds ratio; CI, confidence interval; ATT, after-treatment effect on the treated; ATE, average treatment effect

### Survival Outcomes and Patterns of Failure

**Fig. 1:**
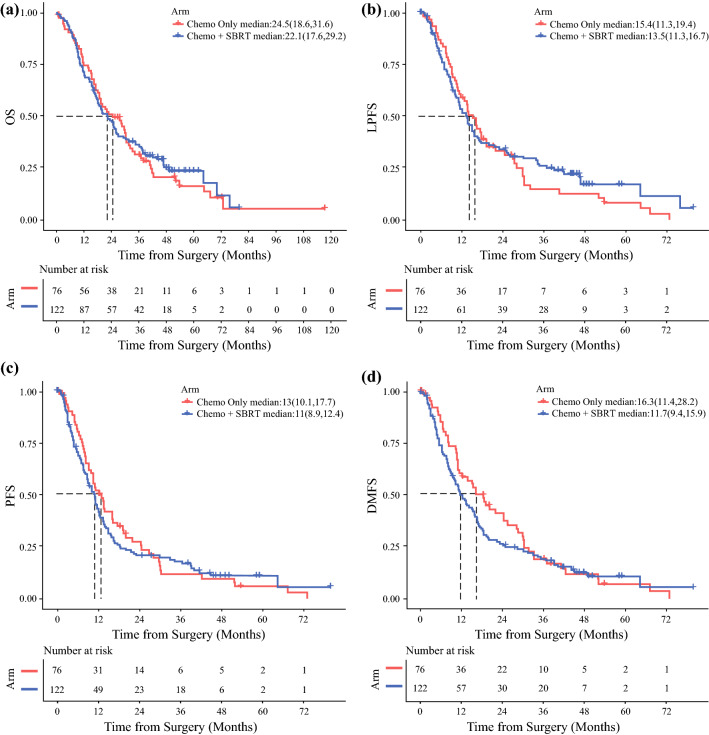
Kaplan-Meier curves of **A** overall survival (OS), **B** progression-free survival (PFS), **C** local progression-free survival (LPFS), and **D** distant metastasis-free survival (DMFS) for the neoadjuvant chemotherapy (nCT) and nCT-SBRT cohorts from the date of surgery. SBRT, stereotactic body radiation therapy

**Fig. 2:**
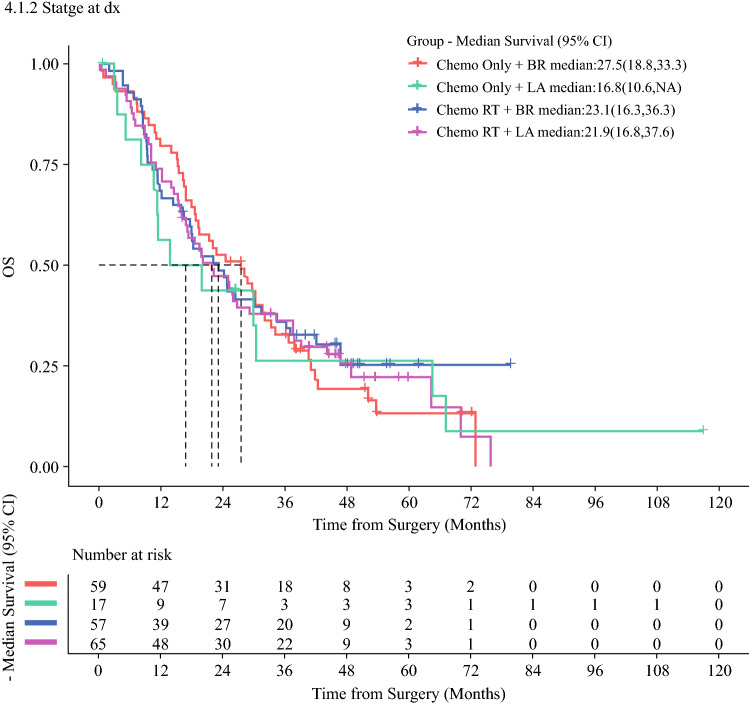
Kaplan-Meier overall survival (OS) curve stratified by induction chemotherapy type and stage from the date of surgery.

Kaplan-Meier analysis showed no significant differences in survival outcomes based on induction therapy type (Table 4; Fig. 1). The median OS after surgery was 22.1 months (range, 17.6–29.3 months) in the nCT-SBRT cohort compared with 24.5 months (range, 18.6–31.6 months) in the CT cohort (*p* > 0.05). After SBRT, the 1-year OS probability was 77.0% (95% confidence interval [CI], 69.9–84.9%) and the 2-year OS probability was 50.4% (95% CI, 42.0–60.2%) for the nCT-SBRT cohort. The LAPC patients treated with nCT-SBRT trended toward a better OS than the patients treated with nCT alone (*p* > 0.05; Fig. 2). In the nCT cohort, 28 patients (36.8%) were still alive 36 months after diagnosis compared with 47 patients (38.5%) in the nCT-SBRT cohort, with 3-year OS probabilities of 31.4% (95% CI, 20.6–42.2%) and 35.9% (95% CI, 27.3%–44.4%), respectively (*p* > 0.05).

**Table 4:** Survival and failure outcomes

	Chemotherapy	Chemotherapy and SBRT	*p* Value
Median survival outcomes: months (95% CI)			
OS	24.5 (18.6–31.6)	22.1 (17.6–29.2)	0.795
PFS	13.0 (10.1–17.7)	11.0 (8.9–12.4)	0.665
LPFS	15.4 (11.3–19.4)	13.5 (11.3–16.7)	0.554
DMFS	16.3 (11.4–28.2)	11.7 (9.4–15.9)	0.331
Failure patterns: *n* (%)			0.306
Patients with imaging follow-up after surgery (*n*)	64	119	
Local recurrence	17 (26.6)	25 (21)	
Distant recurrence	15 (23.4)	36 (30.3)	
Local and distant recurrence	9 (14.1)	26 (21.8)	

SBRT, stereotactic body radiation therapy; CI, confidence interval; OS, overall survival; PFS, progression-free survival; LPFS, local progression-free survival; DMFS, distant metastasis-free survival

The median PFS was 13.0 months (range, 10.1–17.7 months) in the nCT cohort versus 11.0 months (range, 8.9–12.4 months) in the nCT-SBRT cohort (*p* > 0.05). The patterns of failure did not differ significantly between the two groups (*p* = 0.306; Table 4). The site of first failure was local in 27% of the nCT cohort versus 21% of the nCT-SBRT cohort. Distant first failure occurred in 23% of the nCT cohort versus 30% of the nCT-SBRT cohort. Synchronous failure occurred for 14% of the nCT cohort versus 22% of the nCT-SBRT cohort. No significant differences in LPFS (15.4 vs 13.5 months; *p* > 0.05) or DMFS (15.4 vs 11.7 months; *p* = 0.335) were observed.

Multivariable analysis with IPTW demonstrated no significant differences in OS, PFS, LPFS, or DMFS by cohort (Table 3). However, when the patients surviving longer than 36 months were compared with those who had a shorter survival, the patients who survived less than 36 months more frequently had positive margins (37.5% vs 17.9% in nCT [*p* = 0.037]; 12.0% vs. 2.1% in nCT-SBRT [*p* = 0.025]) as well as nodal positivity (67% vs 39% in nCT [*p* = 0.01]; 50.7% vs 27.7% in nCT-SBRT [*p* = 0.03]), and the differences were significant. No patients in the nCT cohort achieved a pCR, but this was significantly associated with long-term survivors in the nCT-SBRT cohort (4.0% in < 36 months vs 12.8% in ≥36 months; *p* = 0.039). Survival outcomes based on margins, nodal status, and pCR are demonstrated in Fig. S1A–C.

### Perioperative Morbidity

Perioperative toxicity was available for 72 nCT patients and 122 nCT-SBRT patients after surgery. The readmission rates and 90-day post-surgery mortality rates did not differ significantly between the cohorts, as demonstrated in Table 5.

**Table 5:** 90-Day perioperative toxicity outcomes

	Chemotherapy *n%*		Chemotherapy and SBRT *n%*		*p* Value*
Perioperative toxicity					0.71
Delayed gastric emptying	15	21.1	21	17.8	
Postoperative pancreatic fistula	10	14.1	11	9.3	
Intra-abdominal abscess	3	4.2	10	8.5	
Bleeding events due to surgery	2	2.8	3	2.5	
Bleeding event due to SBRT	0	0	4	3.4	
Bleeding events not due to SBRT/surgery	0	0	2	1.7	
Wound complications	16	22.5	20	16.9	
Sepsis	1	1.4	7	5.9	
Chyle leak	2	2.8	9	7.6	
Small bowel obstruction	4	5.6	3	2.5	
Biliary leakage	0	0	3	2.5	
Duodeno or gastrojejunostomy leak	0	0	0	0	
Enterocutaneous fistula	0	0	0	0	
Mesenteric venous thrombosis	0	0	1	0.8	
Urinary tract infection	3	4.2	4	3.4	
* Clostridium difficile* colitis	2	2.8	6	5.1	
DVT/PE	2	2.8	4	3.4	
Respiratory complications	3	4.2	4	3.4	
Renal failure	1	1.4	1	0.8	
Cardiac event	4	5.6	5	4.2	
Cerebral vascular event	1	1.4	0	0	
Need for repeat surgery	3	4.2	4	3.4	
90-Day mortality	2	2.8	2	1.7	0.62
Clavien-Dindo classification					
No	30	42.3	52	44.1	
Grade 1	12	16.9	18	15.3	
Grade 2	16	22.5	29	24.6	
Grade 3a	8	11.3	12	10.0	
Grade 3b	3	4.2	6	5.1	
Grade 4a	2	2.8	1	0.8	
Grade 4b	0	0.0	0	0.0	
Grade 5	0	0.0	0	0.0	
Total grade ≥3	13	18.3	19	15.8	0.81

SBRT, stereotactic body radiation therapy; DVT, deep venous thrombosis; PE, pulmonary embolism

Complications were graded according to the Clavien-Dindo classification, and the rate of grade 3 or greater toxicity did not differ significantly between the cohorts, with 11 events (15.3%) in the nCT cohort versus 15 events (12.3%) in the nCT-SBRT cohort (*p* = 0.81). Bleeding events occurred for two patients in the nCT cohort, with one patient requiring an embolization for a bleed from the right hepatic artery (RHA) and another patient with a gastroduodenal artery (GDA) bleed that was conservatively managed. In the nCT-SBRT cohort, bleeding events occurred for seven patients, including four patients who underwent embolization for bleeds from the GDA, RHA, common hepatic artery, and celiac artery, respectively. A fifth patient underwent placement of a stent for a GDA bleed. An additional patient experienced a bleed from the cystic plate, but this event was thought to be related to an irreversible electroporation procedure and was managed conservatively. An additional patient was admitted for a bleed from an upper gastrointestinal source at an outside hospital, but detailed records for this event with respect to the source and type of interventions received were not available.

## Discussion

In a large cohort of BRPC and LAPC patients treated at a high-volume institution, neoadjuvant CT-SBRT significantly improved pathologic outcomes (> 20% increase in the margin-negative resection rate) compared with neoadjuvant chemotherapy alone. Furthermore, the rate of grade 3 or greater complications according to the Clavien-Dindo classification did not differ significantly between the cohorts. Bleeding events did not differ significantly between the cohorts, although a marginal increase in the SBRT cohort cannot be ruled out and warrants further study. Despite the significantly higher percentage of LAPC patients in the nCT-SBRT cohort, survival outcomes did not differ between the two cohorts from the date of surgery.

The role of radiation for patients with localized pancreatic cancer remains controversial. In the locally advanced setting, several historical randomized controlled trials (RCTs) have shown mixed results with the addition of either upfront or consolidative radiation to chemotherapy, but the antiquated techniques and chemotherapeutic agents used in these studies render their applicability to modern day practice questionable.^22–25^

The most modern RCT was LAP07, in which LAPC patients were treated with upfront gemcitabine for four cycles and those without progression were subsequently randomized to two additional cycles of gemcitabine or consolidative CRT.^19^ Although LAP07 was negative for the primary end point of OS, the patients in the CRT cohort did experience significantly improved local control (32% vs 46%).^19^ Given that systemic therapy was gemcitabine alone, systemic control was poor, with 60% of the patients eligible for randomization to CRT due to progression.^19^ Furthermore, less than 5% of the patients in the study were surgically explored, which prevented the ability to assess the role of radiation for margin sterilization and local recurrence risk reduction in LAPC.^19^

Since LAP07, several reports from high-volume institutions, including ours, have shown much higher rates of resection and increased OS in the setting of multi-agent chemotherapy regimens such as FOLFIRINOX.^3–5,26–29^ Notably, these reports describe patients who also have been treated nearly universally with neoadjuvant radiation therapy. High rates of margin sterilization in the locally advanced setting after chemotherapy alone has yet to be demonstrated, but the inferior margin-negative resection rate of 69.7% suggests the added value of radiation in this context.^4,26–29^

Newer studies recently in which patients received FFX followed by CRT have reported favorable R0 resection rates, ranging from 88% to 100%.^3,30–32^ One study compared FFX followed by CRT or chemotherapy alone, and as in our study, outcomes were improved with the addition of CRT.^33^ A single-arm study of 50 patients in Italy with neoadjuvant FFX and SBRT reported that SBRT delivery to the 39 patients without progression after FFX was associated with a significantly improved median OS (18 months in the SBRT group vs 5 months in the non-SBRT group).^34^ After SBRT, seven patients had an RO resection and experienced a significantly better 3-year OS than non-resected patients (43% vs 6.5%).^34^

In a recent phase 1 trial in the Netherlands, 50 patients were treated with eight cycles of FFX followed by SBRT.^35^ Due to progression or toxicity with FFX, only 78% of the patients proceeded to receive SBRT. Six patients who underwent a resection after FFX or SBRT had R0 resection with a complete pCR.^34^ The median OS for these six patients after resection was 23 months (95% CI, 13–34 months).^35^

Reports on the use of CRT in the borderline resectable setting have demonstrated improved pathologic outcomes and in some studies improved OS that mimics resectable disease in those able to undergo subsequent surgery. Although the patient numbers were small, an RCT from Korea exploring upfront CRT versus upfront surgery in BRPC was terminated early due to a much higher rate of margin sterilization in the CRT cohort.^17^ Similarly, the PREOPANC study randomized both resectable and BRPC patients to upfront surgery versus gemcitabine-based CRT.^18^ Although the study did not show a difference for its primary endpoint of OS for the entire cohort with the use of neoadjuvant chemoradiation, a dramatic difference was seen in margin sterilization favoring the CRT cohort (70% vs 40%; *p* < 0.001), and more importantly, a significant improvement was seen in the LPFS and PFS intervals. Furthermore, in the subset of BRPC patients, OS was improved in the CRT cohort compared with upfront surgery.

Of course, these results raise the question concerning the value of preoperative radiation in the setting of neoadjuvant multi-agent systemic therapy, which was explored in two studies led by the Alliance consortium. Specifically, Alliance A021101 was a single-arm study evaluating FFX followed by CRT for BRPC patients. The median OS in this study was 21.7 months, and 15 (68%) of 22 patients underwent resection with a margin-negative resection rate of 93%.^36^ The resected patients experienced a significantly better 18-month OS than the non-resected patients (67% vs 43%).^36^ Importantly, all the patients were treated at high-volume pancreatic centers, and 73% required vessel reconstruction.

A subsequent study, Alliance 021501, was designed in a randomized fashion to explore the additive value of radiation beyond neoadjuvant FFX alone.^37^ In this study, hypofractionated radiation therapy (SBRT, 40 Gy in 5 fractions or 25 Gy in 5 fractions if constraints were not made due to anatomy) was administered. A higher proportion of patients in the SBRT cohort failed to undergo R0 resections, resulting in inferior OS.^37^ Although the exact cause was unclear, the SBRT cohort had significantly higher CA 19-9 markers at baseline, had metastases develop in a higher proportion of patients before or at the time of surgery, and had a higher proportion of patients who did not undergo surgery despite localized disease likely due to acute inflammation attributed to radiation therapy. Because only 40 patients in this study received SBRT, it was not powered to definitively determine the role of SBRT in this setting, but it did suggest that the addition of SBRT adds a level of complexity that may cause some challenges during surgery. This was evidenced by the fact that only 19 of the patients (45%) underwent resection and only 14 of the patients (74% of the resected patients; 35% of the patients treated with radiation therapy) underwent R0 resection.^37^ Nevertheless, pathologic outcomes were improved with SBRT, suggesting that well-selected patients are likely to derive benefit from SBRT and subsequent surgery.^37^

Although the pathologic outcomes, including the R0 resection rate, were significantly improved with SBRT in our study, the survival outcomes did not differ between our cohorts. Although improvements in systemic control certainly will have the greatest impact on survival outcomes, local failure rates are not trivial, as demonstrated by the fact that more than 40% of the patients in both cohorts experienced local failure as part of the pattern of the first failure. We believe this highlights the need to continue refining and intensifying the manner in which radiation is delivered for patients with BRPC and LAPC. Dose escalation and modification of field design may represent pathways to achieve this goal. Indeed, in the LAPC setting, data suggest improved outcomes if a higher radiation dose can be safely administered, but the radiation tolerance of nearby stomach and bowel structures remains limiting.^38,39^

These aforementioned data have primarily been in the non-operative setting, but such dose escalation may also be applicable in the neoadjuvant setting. Certainly, dose escalation has shown tremendous improvement in local outcomes at other adenocarcinoma sites that are more conducive to safe administration of “ablative” doses, such as lung and liver tumors.^40,41^ As advances in radiation technologies (motion management, enhanced image guidance, and adaptive therapy) have improved the precision of radiation delivery, dose-escalation studies attempting to deliver ablative doses are underway, but novel strategies to allow dose escalation likely will be needed.^42–44^ Furthermore, the radiation target volume itself (tumor margin and coverage of elective nodal regions) remains non-standardized and likely considerably heterogeneous in practice. Although gross disease plus a 3- to 5-mm margin has traditionally been the target for SBRT, in the preoperative setting it is possible that peri-pancreatic perineural tracts and lymphatic channels represent the more important target.^45–51^

Several limitations of this study must be acknowledged, such as the retrospective, single-institution nature of the study and the associated potential for bias. For example, although balanced between cohorts, the utilization of multiagent chemotherapy regimens were nonetheless different, and the study could not fully control for this difference. Notably, the imbalance of LAPC patients between treatment arms was more likely to have influenced outcomes negatively in the nCT-SBRT cohort, but it also should be noted that resectability staging (BRPC vs LAPC) may not necessarily be clearly associated with differences in survival outcomes. Additionally, selection bias may have included the decision to administer neoadjuvant radiation to patients with a less robust response to neoadjuvant chemotherapy alone. In addition, patients found to have metastatic disease with restaging after induction therapy before surgery or at the time of surgical exploration would have been excluded from this analysis. Furthermore, we did not have data available to assess the proportion of patients at our institution who received either nCT or nCT-SBRT and could not proceed to surgical exploration.

Finally, although the study was focused on pathologic outcomes, clinical outcomes such as differences in local progression-free survival, overall progression-free survival, and ultimately overall survival represent the more important end points. Certainly, exploration of ways in which radiation can be refined to influence such outcomes should be the subject of future study.

## Conclusion

Despite a significantly larger proportion of patients with LAPC, those treated with nCT-SBRT were more likely to have a robust pathologic response and undergo margin-negative resection than those treated with nCT alone. Although the nCT-SBRT cohort received less adjuvant therapy than the nCT cohort, and although 30% of the nCT patients received adjuvant chemoradiation, the survival outcomes still were similar in both cohorts. More data are needed to refine the determination of which patients benefit from neoadjuvant SBRT and how radiation administration can be further optimized to influence locoregional control, limit toxicity, and improve survival outcomes.

### Electronic supplementary material

Below is the link to the electronic supplementary material.

Supplementary file1 (DOCX 1305 KB)

## Appendix 1

Patient eligibility and follow-up evaluation. Patients were eligible for inclusion in this study if they had each of the following: a histologic diagnosis of pancreatic adenocarcinoma, a radiographic diagnosis of borderline resectable pancreatic cancer (BRPC) or locally advanced pancreatic cancer (LAPC) per the National Comprehensive Cancer Network (NCCN) guidelines,^51^ neoadjuvant therapy before surgical resection, surgical exploration, and sufficient follow-up evaluation, defined as two or more clinical encounters after surgery. Clinical follow-up evaluation for the patients in the study consisted of a history as well as a physical and pancreatic protocol computed tomography scan, initially obtained at 3-month intervals after surgery, with subsequent intervals determined by the clinical team. Cancer antigen (CA) 19-9 levels also were generally obtained at follow-up visits but at the discretion of the clinical team.

## Appendix 2

Stereotactic body radiation therapy (SBRT) planning and treatment objectives. In brief, after placement of fiducials in the pancreatic tumor under endoscopic ultrasound guidance, patients in supine position with arms up underwent a computed tomography simulation with intravenous contrast and immobilization using a Vac-lok (CIVCO Medical Solutions, Coralville, IA, USA) or Alpha-cradle (CIVCO Medical Solutions, Coralville, IA, USA). Patients were treated to a dose of 33 Gy (10–15% heterogeneity allowed) in five fractions on five consecutive weekdays. Motion management was most commonly addressed using active breathing control (ABC; Elekta, Stockholm, Sweden), although a minority of patients were treated under a free-breathing approach using a customized internal tumor volume expansion based on assessment with a four-dimensional computed tomography scan. The target volume included gross disease as well as the full circumference of the involved vasculature at the level of involvement. Planning tumor volume (PTV) margin expansion was 2 to 3 mm. After SBRT, the patients in both cohorts received further systemic therapy before and after surgery at the discretion of the medical oncologist. A subset of patients in the neoadjuvant chemotherapy (nCT) cohort with positive margins after surgery received adjuvant radiation with conventional fractionation (25–30 fractions, often with concurrent capecitabine).

## Appendix 3

Statistical analysis. Overall survival was recorded as the time from the end of surgery to death. The date of death was sourced from medical records and the Social Security Death Index. If the date of death was not available, survival was censored at the date of the last recorded clinical encounter. Progression-free survival (PFS) was measured as the interval from the end of surgery to the time of the first radiographic evidence of failure or death, whichever occurred earlier, and censored at the date of the last recorded imaging follow-up evaluation. Local progression-free survival (LPFS) and distant metastasis-free survival (DMFS) were recorded as the time for the first occurrence of locoregional or distant failure, respectively, or death, whichever occurred earlier. Overall survival (OS), progression free-survival (PFS), local PFS (LPFS), and distant metastasis-free survival (DMFS) were estimated using Kaplan-Meier analysis and compared with the log-rank test. Clinical and treatment covariates were compared between the two patient cohorts using Fisher’s exact test or Student’s *t* test. The Cox regression model was used to estimate the hazard ratio (HR) between the two cohorts as well as other prognostic factors, with the corresponding 95% confidence interval (CI). The inverse probability of treatment weighting (IPTW) using the propensity score was applied to address confounders in comparing the cohorts for pathologic and survival outcomes and assessing the effect of adjuvant chemotherapy within each cohort. Age at diagnosis, sex, National Comprehensive Cancer Network (NCCN) stage at diagnosis, tumor location, pre- and post-neoadjuvant chemotherapy CA19-9, baseline Eastern Cooperative Oncology Group (ECOG), and duration and type of neoadjuvant chemotherapy in each cohort were used to calculate the propensity score using generalized boosted regression model.^52^ Cancer antigen 19-9 (CA 19-9) measurements and ECOG were not available for all the patients. Multiple imputation by chained equations (MICE) was used to account for missing data.^53^ All the clinical and treatment covariates presented in Table 1 were included in the multiple imputation model. Average treatment effect (ATE) and average treatment effect on treated (ATT) were estimated using logistic regression or Cox regression (for survival outcomes) with the IPTW in each of 20 imputed datasets and then pooled using Rubin’s rules.^54,55^ A two-sided significance level of 0.05 was used throughout. Statistical analyses were performed using R 4.0.1. (R Foundation for Statistical Computing, Vienna, Austria.)^56,57^
