# Supplementary material for: Neoadjuvant Stereotactic Body Radiotherapy After Upfront Chemotherapy Improves Pathologic Outcomes Compared With Chemotherapy Alone for Patients With Borderline Resectable or Locally Advanced Pancreatic Adenocarcinoma Without Increasing Perioperative Toxicity
Source: Ann Surg Oncol. 2022 Feb 7;29(4):2456–68. doi: 10.1245/s10434-021-11202-8 (PMC8933354; doi:10.1245/s10434-021-11202-8)

**Supplementary Figure 1A-1C:** Kaplan-Meier OS curve from the date of diagnosis stratified by 1A) margin status and treatment cohort, 1B) nodal status and treatment cohort, and 1C) pathological complete response and treatment cohort.


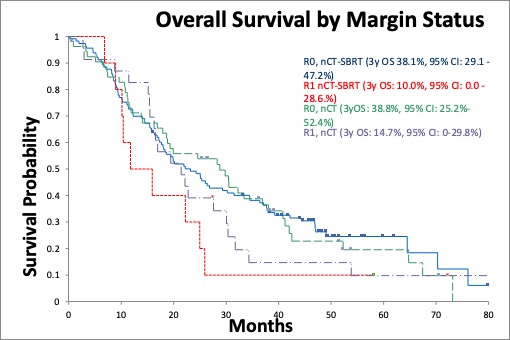


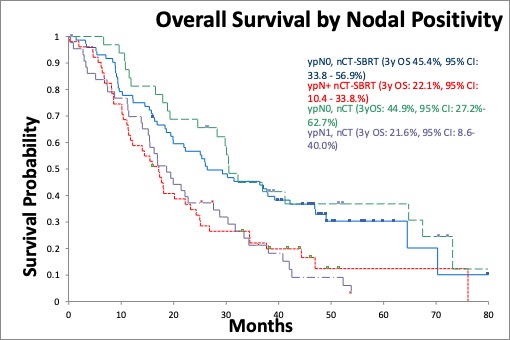


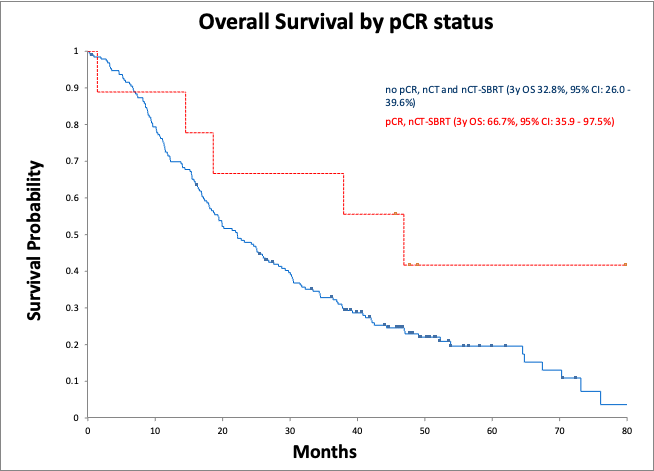

Supplement: Supplementary file 1 — Supplementary file1 (DOCX 1305 KB) [file 10434_2021_11202_MOESM1_ESM.docx]
